# Chemical Composition, Antioxidant Capacity, and Anticancerous Effects against Human Lung Cancer Cells of a Terpenoid-Rich Fraction of *Inula viscosa*

**DOI:** 10.3390/biology13090687

**Published:** 2024-09-02

**Authors:** Fatiha Seglab, Mazen Abou Assali, Thoraya AlYafei, Hassan Hassan, Diana C. G. A. Pinto, Safaa Baydoun, Asmaa A. Al Thani, Abdullah A. Shaito

**Affiliations:** 1 Biomedical Research Center, Qatar University, Doha P.O. Box 2713, Qatar; 2 Environmental Science Center, Qatar University, Doha P.O. Box 2713, Qatar; 3 LAQV-REQUIMTE & Department of Chemistry, University of Aveiro, Campus de Santiago, 3810-193 Aveiro, Portugal; diana@ua.pt; 4 Department of Biological Sciences, Faculty of Science, Beirut Arab University, Beirut P.O. Box 11-5020, Lebanon; 5 Department of Biomedical Sciences, College of Health Sciences and Basic Medical Sciences, College of Medicine, Qatar University, Doha P.O. Box 2713, Qatar

**Keywords:** *Inula viscosa*, *Dittrichia viscosa*, lung cancer, A549 cells, antioxidant capacity, cytotoxic activity, terpenoids, dichloromethane

## Abstract

**Simple Summary:**

*Inula viscosa* (synonymous of *Dittrichia viscosa*) is a plant used in traditional Mediterranean and Middle Eastern medicine to manage various illnesses. While it has shown anticancer effects against various cancer cell lines, its impact on lung cancer has not been under extensive study. This research explored the potential of the methanolic and aqueous extracts and a terpenoid-rich fraction of *I. viscosa* leaves and stems against human lung cancer cells. We found that the methanolic extract of *I. viscosa* leaves was particularly effective in reducing the viability of lung cancer cells in vitro, and other cancer cell types, without affecting normal cells. The terpenoid-rich fraction demonstrated anticancer properties by attenuating the levels of proliferation marker proteins, activating the intrinsic apoptosis pathway, and inhibiting cell migration. GC/MS analysis revealed that the terpenoid-rich fraction encompasses several metabolites that can substantiate its biological activities.

**Abstract:**

*Inula viscosa* is a widely used plant in traditional Mediterranean and Middle Eastern medicine for various illnesses. *I. viscosa* has been shown to have anticancer effects against various cancers, but its effects against lung cancer have been under limited investigation. At the same time, *I. viscosa* is rich in terpenoids whose anti-lung cancer effects have been poorly investigated. This study aimed to examine the potential anticancer properties of methanolic and aqueous extracts of stems and leaves of *I. viscosa* and its terpenoid-rich fraction against human lung cancer A549 cells. Results showed that the methanolic extracts of *I. viscosa* had significantly higher polyphenol and flavonoid content and radical scavenging capacity than the aqueous extracts. In addition, leaves methanolic extracts (IVLM) caused the highest reduction in viability of A549 cells among all the extracts. IVLM also reduced the viability of human ovarian SK-OV-3, breast MCF-7, liver HepG2, and colorectal HCT116 cancer cells. A terpenoid-rich *I. viscosa* fraction (IVL DCM), prepared by liquid-liquid separation of IVLM in dichloromethane (DCM), displayed a substantial reduction in the viability of A549 cells (IC_50_ = 27.8 ± 1.5 µg/mL at 48 h) and the panel of tested cancerous cell lines but was not cytotoxic to normal human embryonic fibroblasts (HDFn). The assessment of IVL DCM phytochemical constituents using GC-MS analysis revealed 21 metabolites, highlighting an enrichment in terpenoids, such as lupeol and its derivatives, caryophyllene oxide, betulin, and isopulegol, known to exhibit proapoptotic and antimetastatic functions. IVL DCM also showed robust antioxidant capacity and decent polyphenol and flavonoid contents. Furthermore, Western blotting analysis indicated that IVL DCM reduced proliferation (reduction of proliferation marker Ki67 and induction of proliferation inhibitor proteins P21 and P27), contaminant with P38 MAP kinase activation, and induced the intrinsic apoptotic pathway (P53/BCL2/BAX/Caspase3/PARP) in A549 cells. IVL DCM also reduced the migration of A549 cells, potentially by reducing FAK activation. Future identification of anticancer metabolites of IVL DCM, especially terpenoids, is recommended. These data place *I. viscosa* as a new resource of herbal anticancer agents.

## 1. Introduction

Lung cancer is a leading cause of cancer incidence and death globally, with approximately two million new cases and 1.8 million estimated fatalities annually [1,2,3]. Therapies for lung cancer include platinum-based chemotherapy, surgical resection, radiotherapy, combination chemotherapy, and targeted immunotherapy, depending on the cancer stage, progression, or the presence of metastasis. Despite significant advancements in cancer therapies over recent years, platinum-based chemotherapy remains the mainstay of treatment for patients with advanced non-small cell lung cancer (NSCLC) [4,5]. Response rates to this chemotherapy range between 25 and 35%, the median survival lingers between 8 and 12 months, and only 30 to 40% of patients reach the one-year survival mark [6]. Notably, the combination of platinum-based chemotherapy with other therapeutic modalities is pivotal for the management of advanced lung cancer cases. Nevertheless, therapies for lung cancer suffer from a spectrum of side effects and unsatisfactory success rates due to inadequate response, thereby immensely impacting patients’ quality of life and limiting the range of dosages that can be safely administered. Importantly, resistance to therapy and the resulting tumor recurrence are the significant challenges facing existing lung cancer therapeutic modalities [7]. All these limitations underscore the urgency to discover and develop innovative anticancer therapies for lung cancer. In this context, herbal medicine and plant-based therapies may provide promising alternatives.

Plant-derived natural products have varied chemical structures, low toxicity levels, widespread availability, and cost-effective production and therefore present an encouraging avenue for creating novel and potent anticancer treatments that target the hallmarks of cancer. Furthermore, the use of plant-derived metabolites as an adjuvant therapy in combination with conventional treatments will be a promising approach to enhance cancer treatment practices in the near future [8].

Currently, there is a revived interest in drug discovery using plants, offering new opportunities in the search for cancer therapies. Plant-derived therapeutic remedies usually exhibit multifaceted therapeutic effects and act on multiple targets implicated in several cellular mechanisms and molecular pathways driving cancer incidence or progression. Plant extracts and their metabolites have been reported to act on the cell cycle and its regulatory proteins [8,9,10], exhibit proapoptotic [8,9,10] and antimetastatic properties [8,9], induce autophagy [8,9], scavenge reactive oxygen species (ROS) [8,9], impair angiogenesis [8,9,10], modify epithelial to mesenchymal transition (EMT) [8,9], target drug resistance pathways [8,9,11], modulate the expression and activity of matrix metalloproteinases (MMPs) [8], among other roles [8,9,10,11]. Considering the vast potential of plants as a source of chemotherapeutic agents, this study focused on the anti-lung cancer effects of the plant *Inula viscosa* (L.) Aiton.

*Inula viscosa* (L.) Aiton (*Asteraceae* family), now accepted as *Dittrichia viscosa* Greuter and commonly known as *Inula*, is a perennial evergreen, highly branched herbaceous plant with sticky leaves and yellow flowers [12,13]. *I. viscosa* is a native widespread plant in Mediterranean and North African countries and an invasive plant in Australia and North America [12,14]. *I. viscosa* has been a heavily indicated plant in traditional medicine for decades to treat numerous ailments, including hypertension, gastrointestinal disorders, microbial infections, skin diseases, diabetes, inflammatory diseases, cancer, and wound healing [13,15]. Recent research confirms that *I. viscosa* extracts and essential oils have a variety of biological properties, including antipyretic [16], analgesic [15], antiviral [17], antibacterial [18], antifungal [18], antiproliferative [13], antidiabetic [19], neuroprotective [12], and antioxidant [13,15] qualities, aligning with the ethnomedicinal uses of the plant. Relatedly, *I. viscosa* has been reported to have antineoplastic properties against numerous human cancer cell lines [20], including non-Hodgkin Burkitt lymphoma [21], breast [22,23], colorectal [24,25,26], cervical [27], melanoma [28], and skin cancer cell lines [29], among others [15]. Moreover, the plant has shown anticancer activity in vivo against colorectal [24] and skin cancer [29] in mouse models. Nevertheless, the anti-lung cancer properties of *I. viscosa* are understudied [30].

Numerous studies aiming to identify the bioactive phytochemicals responsible for the biological properties of the plant were performed and demonstrated that *I. viscosa* is indeed rich in metabolites belonging to flavonoids, other polyphenols, and terpenoids [15,22,30,31,32,33,34]. The plant also contains lignins, sesquiterpene lactones and acids, steroids, and alkaloids [31,32,35]. Extracts and essential oils of *I. viscosa* are especially rich in terpenoids, including monoterpenes, oxygenated monoterpenes, sesquiterpenes, oxygenated sesquiterpenes, diterpenes, and triterpenes [15,36,37].

Terpenoids/terpenes are greatly functionalized natural products derived from the isoprenoid precursor isopentenyl diphosphate (IPP). With more than 70,000 terpenoids identified so far, terpenoids are the most prevalent secondary metabolites in nature, being synthesized by a vast range of plants, fungi, and bacteria [38,39]. They have diverse functions in plants, including localization in the cell membrane, acting as pheromones to attract pollinators, repelling herbivores, fighting parasites and pathogens, and many other functions [38,39]. Terpenoids are utilized in many modern applications, including industrial (synthesis of fuels, food flavorings, extraction solvents) and pharmaceutical products [38]. Terpenoids exhibit antiviral, antimicrobial, antifungal, cytotoxic, and anti-inflammatory properties [38,40,41]. Of specific interest to this study, terpenoids have demonstrably exhibited cytotoxic and anticancer effects against several human cancer cell lines, and some terpenoids have demonstrated in vivo anticancer activity in mice models [41,42,43,44]. With the urging quest for more novel drugs of botanical origin, there is a need to investigate the anticancer effects of terpenoids of ethnobotanical plants widely used in traditional medicine. In this regard, the anticancer effects of *I. viscosa* terpenoids are still understudied.

Considering the reported therapeutic potential of *I. viscosa* and the scarcity of studies on the effects of its terpenoids against cancer, particularly lung cancer, here we have prepared a terpenoid-rich fraction of *I. viscosa* and tested its anticancer properties against human lung adenocarcinoma A549 cells.

## 2. Materials and Methods

### 2.1. Collection and Identification of Plant Material

Fresh aerial parts of *I. viscosa* were randomly collected in November 2022 from At-Tiri village in the South of Lebanon (33°08′02.3″ N; 35°23′34.4″ E). Plant identification was confirmed by Mohammad Al-Zein, a resident plant taxonomist at the American University of Beirut Herbarium, according to index Kewensis as Kingdom: Plantae; Phylum: Tracheophyta; Class: Magnoliopsida; Order: Asterales; Family: Asteraceae; Genus: Dittrichia Greuter; Species: *Dittrichia viscosa* Greuter; Synonym: *Inula viscosa* (L.) Aiton (World flora online record: https://www.worldfloraonline.org/taxon/wfo-0000059214; accessed on 9 September 2024).

### 2.2. Preparation of Methanolic and Aqueous Crude Extracts

The collected plant material was washed and dried in the dark at room temperature. The dried aerial parts were separated into leaves and stems and finely ground into a powder using a blender. Ten grams of the powdered leaves or stems were extracted by maceration in 100 mL of 80% (*v*/*v*) aqueous methanol or 100% distilled water at room temperature for two days, mixing at 150 rpm on a horizontal orbital shaker. Methanol was evaporated to dryness under reduced pressure at 40 °C using a rotary evaporator. The resulting powder was reconstituted in DMSO to obtain crude methanol extract of stems (IVSM) or leaves (IVLM). IVSM and IVLM were stored at −20 °C until use. Additionally, the macerated aqueous extracts of leaves (IVLaq) or stems (IVSaq) were lyophilized into a fine powder using a freeze dryer. Working stock solutions for in vitro cell culture work were prepared by dissolving stock plant extracts (in DMSO) in cell culture media at a 10 mg/mL concentration, followed by filtration through a 0.22 µm syringe filter. The working stock solutions were further diluted in cell culture media to achieve the desired final experimental concentrations.

### 2.3. Preparation of Terpenoid-Rich Fraction (IVLM DCM)

Liquid-liquid extraction of a plant crude methanolic extract is an effective method to obtain a terpenoid-rich fraction. The crude extract is extracted in a low-intermediate-polarity solvent such as hexane, dichloromethane (DCM), or chloroform, among others, to recover slightly polar terpenoids without polar functional groups [38,45]. Starting with the IVLM methanolic extracts, liquid-liquid extraction of IVLM in DCM was performed to obtain the terpenoid-rich fraction (IVL DCM). Following maceration, the IVLM extract was filtered through Whatman No. 1 filter paper, and the organic solvent (methanol) was evaporated at 45 °C under reduced pressure using a rotary evaporator. The crude extract (mainly aqueous fraction) was then filtered and collected. The crude extract was fractionated by liquid-liquid multi-stage extraction using 3 × 50 mL of DCM to obtain the DCM fraction (IVL DCM). All three organic phases from the multi-stage extraction were pooled, dried using a rotary evaporator, and stored at −20 °C until use.

### 2.4. Determination of Total Phenols and Flavonoids Contents

Total polyphenol content (TPC) was calorimetrically determined using the original oxidation/reduction method described by Singleton and Ross [46], with modifications to adapt to a 96-well plate format. Briefly, 12.5 µL of the diluted plant extracts or fractions were added to 125 µL of a 10X diluted Folin–Ciocalteu reagent (ref 1090010100; Supelco-Sigma-Aldrich, Bellefonte, PA, USA). The mixture was incubated for 3 min to accomplish the oxidation reaction, and 500 µL of 2% saturated sodium carbonate solution were added to neutralize the reaction. The products of the reduced metal oxide thus produced have a blue color, which was quantified using a Cytation 5 reader (BioTek-Agilent Technologies, Winooski, VT, USA) at a wavelength of 765 nm. All samples were analyzed in triplicate. A standard curve was prepared using different concentrations of the reference polyphenol gallic acid (GA). TPC of all plant extracts or fractions was expressed as µg GA equivalents (GAE) per gram of the dry matter used to make the extract (µg GAE/g).

The total flavonoids content (TFC) of extracts and fractions was determined using the spectrophotometric method of Chang et al. [47], with modifications to work in a 96-well plate format. Briefly, 50 µL of the diluted plant extracts or fractions were mixed with 50 µL of 2% AlCl3 (ref 7784-13-6, Research Lab, Mumbai, India) in 80% methanol solution and incubated for 20 min to allow for the formation of a flavonoid-aluminum complex. This complex was identified by its characteristic maximum absorption at 415 nm using the Cytation 5 reader. All samples were analyzed in triplicate. A calibration curve was prepared using different concentrations of the reference flavonoid quercetin (QE). TFC was expressed as μg QE equivalents (µg QE) per gram of the dry matter used to make the extract (µg QE/g). TPC and TFC results are presented as mean values ± standard error of the mean (SEM).

### 2.5. Gas Chromatography/Mass Spectrometry (GC/MS) Analysis

The phytochemical composition of IVL DCM fraction was analyzed using GC/MS via a Shimadzu GC/MS-TQ 8040 NX (Shimadzu, Tokyo, Japan) system attached to a triple quadrupole mass spectrometer. Chromatography was conducted on a Restek RTxi-5 Sil MS (30 m × 0.25 mm ID × 0.25 µm) GC capillary column using an injection volume of one µL. A mixture of helium and argon gases was used as carrier gas at a flow rate of 1.5 mL/min, a pressure of 23.1 KPa, and an average velocity of 0.2 s. The source and interface temperatures were 200 °C and 280 °C, respectively. The initial temperature was set at 80 °C for 2 min, then increased to 250 °C at 20 °C/min, and raised to 280 °C at 15 °C/min (held for 12 min). Identification of phytochemicals in IVL DCM was carried out by comparing the obtained retention time and spectral masses with those of chemical compounds in the database of the National Institute of Standards and Technology (NIST version 20).

### 2.6. DPPH Free Radical Scavenging Assay

The antioxidant capacity of *I. viscosa* extracts and fractions was performed following the methodology outlined by Brand-Williams et al. [48]. Different concentrations (0.25, 50, 100, 150, 250, 350, 450, or 750 μg/mL) of *I. viscosa* extracts or fractions were mixed with an equal volume of a 0.5 mM solution of 2,2-Diphenyl-1-picrylhydrazyl (DPPH; ref D9132, Sigma-Aldrich, St. Louis, MO, USA) in methanol. The mixture was left to react in the dark for 30 min at room temperature, and absorbance was measured at 517 nm using a spectrophotometer. Methanol was used as a blank. The percentage of radical scavenging activity was calculated according to the following formula:

$$\%\;Radical\;scavenging\;activity=\left[\frac{{OD}_{blank}-{OD}_{plant\;extract\;at\;each\;concentration}}{{OD}_{blank}}\right]\times 100$$

Percent radical scavenging activity was plotted versus the concentration of the plant extracts or fractions, and the plotted curves were used to calculate the EC_50_ of inhibition of DPPH free radical production. EC_50_ of the potent antioxidant L-ascorbic acid was measured for comparison.

### 2.7. ABTS Radical Scavenging Assay

The ABTS cation scavenging assay was performed according to the method of Re et al. [49], with modifications. ABTS radical cation (ABTS•) was produced by reacting 20 mL of 7 mM ABTS (ref GE7230, Glentham Life Sciences, Corsham, UK) stock solution (dissolved in water) with 200 µL of 70 mM potassium persulfate aqueous solution. The mixture was allowed to stand in the dark at room temperature for 12–16 h before use. A fresh working solution was prepared by diluting 1 mL of ABTS• solution with the proper amount of distilled water to obtain an absorbance of 0.700 ± 0.02 units at 745 nm. Afterward, 100 µL of this solution were mixed with 10 μL of different concentrations of the plant extracts or fractions and incubated for 15 min in the dark. Absorbance was measured at 745 nm after 15 min. Distilled water and L-Ascorbic acid were used as blank and reference control, respectively.

EC50 values of scavenging of ABTS• by the different plant extracts or fractions were calculated by plotting the % radical scavenging activity versus concentration. Percent cation-scavenging activity was calculated using the following formula.

$$\%\;Cation\;scavenging\;activity=\left[\frac{{OD}_{blank}-{OD}_{plant\;extract\;at\;each\;concentration}}{{OD}_{blank}}\right]\times 100$$

### 2.8. Cell Culture and MTT Cell Cytotoxicity Assay

A549 human lung adenocarcinoma cells, the most common subtype of NSCLC, which accounts for approximately 85% of lung cancer cases [50], human normal neonatal fibroblasts (HDFn), SK-OV-3 (human ovarian cancer), MCF-7 (human breast cancer), MDA-MB-231 (human breast cancer), HepG2 (human liver cancer), and HCT116 (human colorectal cancer) cells were obtained from ATCC and cultured at 37 °C and 5% CO_2_ in a humidified incubator. A549 and MCF7 cells were grown in RPMI media, while HDFn, SK-OV-3, MDA-MB-231, HepG2, and HCT116 cells were grown in DMEM media. Media were supplemented with 10% fetal bovine serum (FBS; Sigma Aldrich) and 1% penicillin/streptomycin (Corning, Bedford, MA, USA). Cells were passaged using Trypsin-EDTA (ref 3920459; VWR International, Lutterworth, Leicestershire, UK). Cell viability was measured using the MTT assay (ref ab211091, Abcam, Cambridge, UK) according to the manufacturer’s instructions. Briefly, cells (5.0 × 10^3^ cells/well of a 96-well cell culture plate) were seeded for 24 h before treatment. The culture medium was then replaced with fresh complete medium containing various concentrations (20, 40, 50, 75, or 100 µg/mL) of *I. viscosa* extracts or fractions. At 24, 48, or 72 h post-treatment, the cells were washed with 1X PBS, and then 100 μL of MTT reagent were added to each well, and the wells were incubated for three hours in the dark at 37 °C and 5% CO_2_.

The formed formazan was dissolved by adding 100 μL DMSO, and the absorbance of the dissolved formazan solution was measured at 540 nm using a Tecan microplate reader (Tecan Group Ltd., Männedorf, Switzerland). Cell viability was calculated as the percent cell viability of the treated cells in comparison with cells treated with an equal concentration of DMSO as in the extract, vehicle-control treated cells, the viability of which was set as 100% viability. Each concentration of the plant extracts or fractions was tested in triplicate. Each experiment was repeated three times (*n* = 3).

### 2.9. DAPI Staining

A549 cells were seeded in separate wells of a 96-well cell culture plate as described for the MTT assay and treated with 40 and 75 µg/mL IVL DCM fraction for 24 h. Cells treated with an equal concentration of DMSO were used as vehicle controls. Cells were washed with 1X PBS, fixed with 100 µL of 4% formaldehyde (*v*/*v* in 1X PBS) for 15min, and washed with 1X PBS. The cells were then stained with 100 µL of 1 µg/mL of DAPI solution (4′-6-diamidino-2-phenylindole; ref D1306, Invitrogen, Carlsbad, CA, USA) for 5 min in the dark. Cells were washed using 1X PBS, and fluorescence was observed and imaged using a BioTek Cytation 5 reader.

### 2.10. Crystal Violet Staining

Cells were seeded in a 6-well plate (2.0 × 10^5^ cells/well) for 24 h and treated with 40 and 75 µg/mL IVL DCM fraction for another 24 h. Cells were washed with 1X PBS, fixed using 4% formaldehyde (*v*/*v* in 1X PBS) for 15 min, and washed with 1X PBS. Cells were then stained with a 1% crystal violet (*v*/*v* in methanol) solution for 15 min in the dark. Finally, the cells were washed and imaged using the Invitrogen™ EVOS^®^ FL Cell Imaging System (Thermo Fisher Scientific, Waltham, MA, USA).

### 2.11. Western Blotting Analysis

A549 cells were seeded in a 10 cm cell culture dish (5.0 × 10^5^ cells/dish) and allowed to grow for 24 h. The cells were then treated with 40 or 75 µg/mL of IVL DCM for 24 h. Subsequently, the cells were washed twice with 1X PBS, lysed using a 1X cell lysis buffer [ref 9803S, Cell Signaling Technology, Inc. (CST), Danvers, MA, USA] containing 1X Roche Complete Protease inhibitor (ref 11697498001; Sigma-Aldrich) for 5 min on ice, scraped, collected, and subjected to centrifugation at 1.4 × 10^4^ rpm for 15 min at 4 °C. Supernatants protein concentration was quantified using Pierce™ BCA Protein Assay Kit (ref A53225; ThermoFisher Scientific, Rockford, IL, USA). An amount of 25 μg of protein lysates was resolved using 10% SDS-PAGE and transferred to a PVDF membrane (Immobilon PVDF; Biorad, Hercules, CA, USA). The membrane was then blocked with 1X blocking buffer (ref 37520, ThermoFisher Scientific) in 1X TBST (1X TBS, 0.1% Tween 20) for 1.5 h at room temperature. Immunodetection was performed by incubating the membrane with specific primary antibodies, diluted in X blocking buffer, at 4 °C overnight. Primary antibodies used were: anti-human poly-adenosine diphosphate (ADP) ribose polymerase (PARP) 46D11 rabbit mAb which can detect the full-length and cleaved forms of PARP (ref 9532 CST, dilution 1/1000), P53 rabbit mAb (ref 2527S CST, dilution 1/500), Ki67 rabbit polyclonal antibody (ref 28074-1-AP, Proteintech, Rosemont, IL, USA, dilution 1/1000), mouse anti-human B-cell lymphoma 2 (BCL2) (ref 15071S CST, dilution 1/1000), rabbit anti-Bcl-2 associated X protein (BAX) (D2E11 ref 5023 CST; dilution 1/1000), P38 MAPK polyclonal antibody (14064-1-AP, Proteintech, dilution 1/1000), phospho-p38 MAPK (Thr180/Tyr182) antibody(ref 9211 CST, dilution 1/1000), Caspase3 antibody (ref 9662 CST, 1/1000 dilution), cleaved Caspase3 (c-Caspase 3; Asp175) 5A1E rabbit mAb (ref 9664 CST, dilution 1/1000), β-actin 8H10D10 mouse mAb (ref 3760 CST, dilution 1/1000), P21 Waf1/Cip1 12D1 rabbit mAb (ref 2947 CST, 1/1000), P27 (CST, 1/1000), phospho-FAK (p-Fak Tyr 397) D20B1 rabbit mAb (ref 8556 CST, dilution 1/1000), FAK D2R2E rabbit mAb (ref 13009 CST, dilution 1/1000). Membranes were then washed with 1X TBST and incubated with a horseradish peroxidase (HRP)-conjugated goat anti-rabbit (ref 7074 CST, dilution 1/1000) or anti-mouse IgG secondary antibody (ref 7076 CST, dilution 1/1000) for 1.5 h at room temperature followed by washing in 1X TBST. Immunoreactive bands were detected using the SuperSignal™ West Pico PLUS Chemiluminescent Substrate kit (ref 34577 ThermoFisher Scientific) and scanned using the Invitrogen™ iBright Imaging System (Thermo Fisher Scientific, Waltham, MA, USA). The intensity of the obtained bands was quantified using ImageJ software version 1.54g (NIH, Bethesda, MD, USA; https://imagej.net/ij, accessed on 31 August 2024). All bands were normalized to β-actin, which was used as a loading control, except for p-P38 and p-FAK, which were normalized to their non-phosphorylated protein forms (Supplementary Materials).

### 2.12. Scratch/Wound-Healing Assay

A549 cells were cultured until confluence to create a monolayer in 6-well plates, then treated with 40 and 75 µg/mL of IVL DCM fraction for 24 h. A scrape was made through the confluent monolayer using a sterile 1000 μL micropipette blue tip. The culture medium was removed, and the cells were washed twice with 1 XPBS to remove cellular debris and incubated at 37 °C in fresh medium in the presence or absence of the indicated concentrations of IVL DCM. Photomicrographs of the scratch were taken using light microscopy at baseline (0 h) and 24 and 48 h later, using Invitrogen EVOS^®^ FL. The cellular migration rate was calculated as the average (in μm) ± SEM of the difference between the wound width at time zero and the corresponding time points.

### 2.13. Statistical Analysis

Results were evaluated for statistical difference by one-way ANOVA followed by Tukey’s post hoc multiple comparisons test to calculate *p* values using GraphPad Prism 9 software (GraphPad Software Inc., San Diego, CA, USA). Half maximal inhibitory concentration (IC50) of cell growth was determined by plotting the MTT data as dose-response curves of percent growth inhibition versus the log of extract concentration using GraphPad Prism 9. GraphPad Prism performed curve fitting of the data by non-linear regression to obtain the IC50 values. Data are presented as mean ± SEM, and a *p*-value of *p* < 0.05 was considered statistically significant.

## 3. Results

### 3.1. Extraction Yield, Total Phenolic Content (TPC), Total Flavonoid Content (TFC), and Antioxidant Capacity of Inula viscosa Leaves and Stems Extracts

Table 1 shows the extraction yield, TPC, TFC, and antioxidant capacity of methanolic and aqueous extracts of leaves and stems of *I. viscosa*. Methanol exhibited better extraction yields than water (Table 1). *I. viscosa* leaves had better extraction yields than stems in both the methanolic and water solvents. Interestingly, methanol was consistently a better extraction solvent of *I. viscosa* polyphenols and flavonoids than water. Methanolic extracts of both stems and leaves showed markedly higher TPC and TFC contents than their aqueous counterparts (Table 1). Leaves had higher TFC and TPC contents than stems in each solvent (Table 1). Importantly, the methanolic extract of *I. viscosa* leaves (IVLM) had the highest TPC and TFC contents, recording the mean values of 726.4 ± 1.1 and 303.3 ± 8.8, respectively (Table 1).

**Figure 1:**
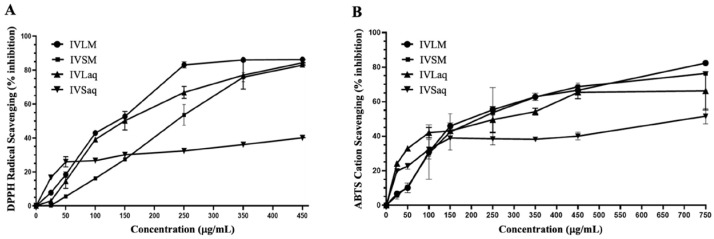
Antioxidant capacities of methanolic and aqueous extracts of *I. viscosa* leaves and stems using the DPPH antioxidant free radical scavenging assay (**A**) and ABTS antioxidant cation scavenging assay (**B**). The graphs were used to calculate EC50 values, as displayed in Table 1.

The antioxidant capacity of *I. viscosa* extracts was measured using DPPH and ABTS antioxidant assays. The antioxidant activity varied substantially depending on the extraction solvent and plant part. IVLM antioxidant capacity in DPPH and ABTS assays was superior to all other extracts regardless of solvent or plant part (Table 1 and Figure 1). IVLM DPPH antioxidant capacity EC_50_ value was 145.7 ± 2.6 µg/mL, followed by the leaves aqueous extract (IVLaq) with an EC_50_ of 155.8 ± 6.0 µg/mL (Table 1 and Figure 1A). The plant part was a determining factor of the DPPH antioxidant scavenging capacity. The leaves showed consistently better radical scavenging capacity than stems in both solvents. Indeed, IVLM and IVLaq showed lower radical scavenging EC_50_ values than IVSM and IVSaq (Table 1 and Figure 1A). Furthermore, methanol was a better extraction solvent than water for each plant part in relation to free radical antioxidant capacity. For stems, the methanolic extract IVSM had a higher antioxidant capacity (EC_50_ = 229.7 ± 3.1 µg/mL) than that of aqueous stems extract IVSaq (EC_50_ = 693.8 ± 3.4 µg/mL), which had the lowest antioxidant capacity of all extracts. For the leaves extract, the methanol extract IVLM antioxidant capacity was slightly higher than the leaves aqueous extract IVLaq (Table 1 and Figure 1A).

The results of the ABTS cation antioxidant assay showed that methanol was a better solvent irrespective of the plant part. IVLM and IVSM (Table 1 and Figure 1B). IVLM had the best ABTS antioxidant capacity (EC_50_ = 236.9 ± 22.2), while IVSM had a comparable capacity (EC_50_ = 239 ± 5.5). IVSaq exhibited the lowest antioxidant capacity of all extracts (EC_50_ = 791 ± 14.5), similar to the results of the DPPH antioxidant capacity (Table 1 and Figure 1B). Moreover, the leaves extracts showed better ABTS antioxidant capacity than stems extracts in both solvents (Table 1 and Figure 1B).

### 3.2. Inula viscosa Methanolic Extracts Reduced the Viability of A549 Lung Cancer Cells

**Figure 2:**
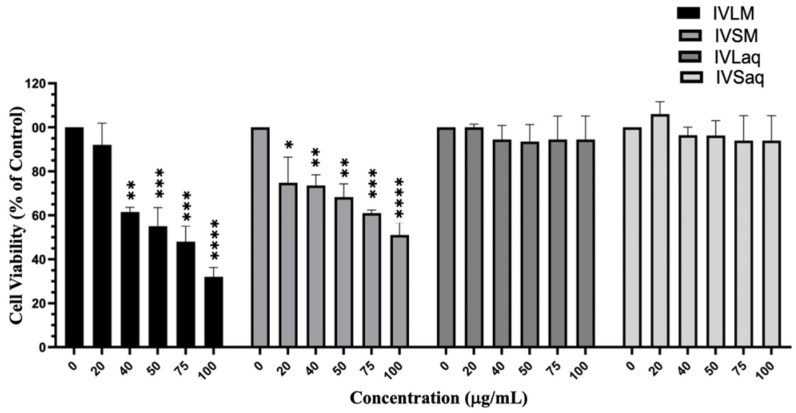
*I. viscosa* leaves and stems methanolic extracts reduced viability of A549 lung cancer cells. A549 cells were treated with *I. viscosa* leaves or stems extracts at the indicated concentrations for 24 h. Cell viability was determined using the MTT assay, and results were expressed as viability percentages compared to control vehicle-treated cells. Data represent the mean ± SEM (*n* = 3). * denotes *p* < 0.05, ** *p* < 0.01, *** *p* < 0.001, and **** *p* < 0.0001.

Aqueous and methanolic extracts of *I. viscosa* stems and leaves were evaluated for their cytotoxic effects against A549 lung cancer cells by the MTT assay. Results revealed that methanolic extracts of both the stems and leaves had noticeably superior cytotoxic effects against A549 cells than their aqueous counterparts. Both VLM and IVSM extracts significantly reduced the viability of A549 cells, while IVLaq and IVSaq did not affect A549 cell viability (Figure 2). Furthermore, IVLM reduced the viability of A549 cells to a higher extent than IVSM (IVLM IC_50_ = 68.7 ± 1.4 µg/mL while IVSM IC_50_ = 97.3 ± 1.3 µg/mL) (Figure 2). IVLM reduced the viability of A549 cells concentration-dependently starting at 20 µg/mL, but the effect was significant (*p* < 0.001) beginning at 40 µg/mL. IVSM also significantly reduced A549 cell viability dose-dependently, starting at the lowest tested concentration of 20 µg/mL (Figure 2).

IVLM also reduced the viability of other human cancer cell lines, including SK-OV-3 (ovarian cancer), MCF-7 (breast cancer), HepG2 (liver cancer), and HCT116 (colorectal cancer) cells (Table 2).

### 3.3. Dichloromethane Fraction of Inula viscosa Leaves Methanolic Extract (IVL DCM) Reduced the Viability of A549 Lung Cancer Cells

Building upon the insights derived from the above findings, IVLM, which demonstrated the best antioxidant capacity, highest TPC and TFC contents, and the most significant cytotoxic properties against A549 cells, was further purified by liquid-liquid separation in DCM to obtain a terpenoid-enriched fraction of *I. viscosa* leaves (IVL DCM).

The cytotoxic effects of IVL DCM fraction were examined in a panel of human cancerous cell lines. Indeed, IVL DCM reduced the viability of several human cancer cell lines, including SK-OV-3, MCF-7, MDA-MB-231 (breast cancer), HepG2, and HCT116 cells (Table 3).

**Figure 3:**
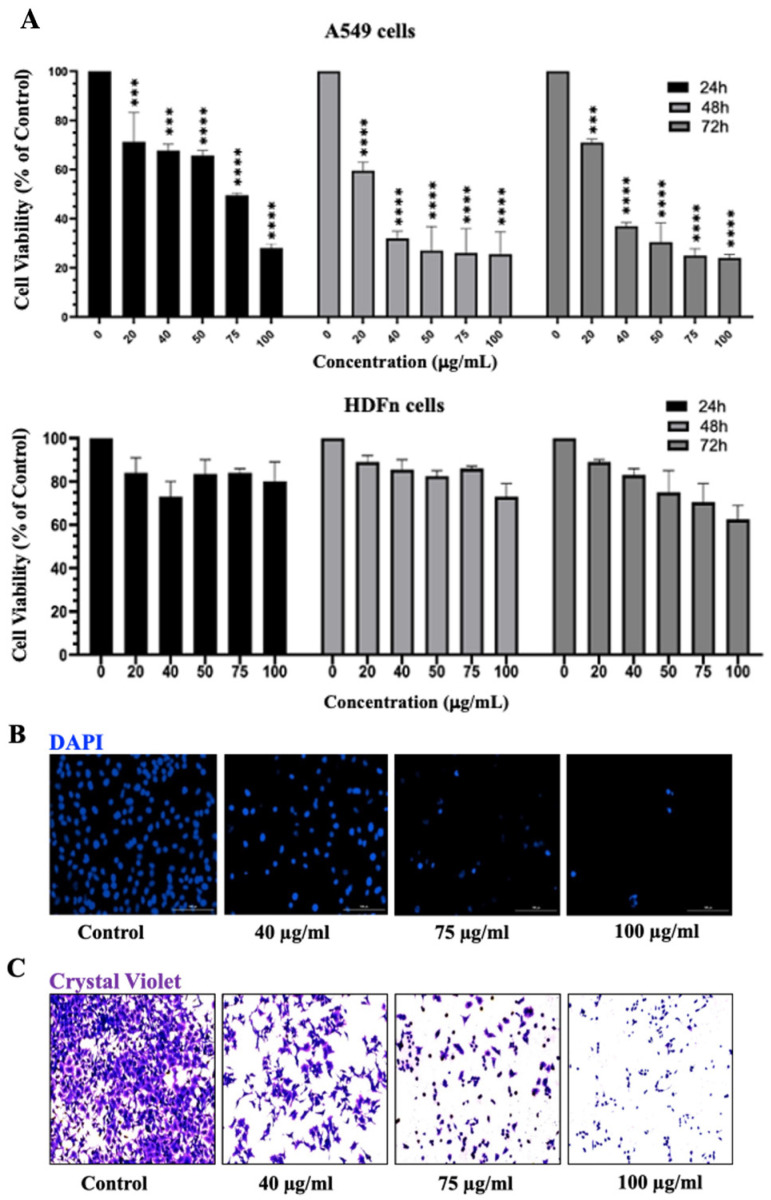
IVL DCM decreased the viability of A549 cells. (**A**) MTT assay was used to measure the viability of A549 and HDFn cells treated with the indicated concentrations of IVL DCM for 24, 48, or 72 h. Data represents the mean ± SEM of the percent viability of treated cells compared to vehicle-treated cells. Data are the mean of three independent experiments (*n* = 3). *** *p* < 0.001, **** *p* < 0.0001. (**B**) A549 cells treated with the indicated concentrations of IVL DCM for 24 h were stained with DAPI nuclear stain. DAPI fluorescence was imaged at 20X magnification using a BioTek Cytation 5 reader. (**C**) A549 cells were treated with the indicated concentrations of IVL DCM for 24 h and then stained with crystal violet. The micrographs represent stained cells imaged at 20X magnification by light microscopy using the Invitrogen EVOS^®^ FL Cell Imaging System.

Moreover, a detailed analysis of the cytotoxic effects of the treatment of A549 lung cancer cells with increasing concentrations (20, 40, 75, and 100 μg/mL) of IVL DCM at 24, 48, and 72 h revealed that IVL DCM significantly attenuated A549 cell viability in a time- and concentration-dependent manner (Figure 3). For instance, cell viability upon treatment with 20, 40, 75, and 100 μg/mL of IVL DCM for 48 h decreased to 72% ± 0.27, 46% ± 0.29, 24% ± 0.09, and 12% ± 0.64 that of vehicle-treated cells, respectively (Figure 3A). IVL DCM IC_50_ values were 70.8 ± 3.7, 27.8 ± 1.44, and 32.1 ± 3.8 μg/mL in A549 cells at 24, 48, and 72 h, respectively (Figure 3A). Interestingly, IVL DCM did not reduce the viability of normal human neonatal fibroblasts (HdFn cells), hinting that IVL DCM may have selectivity towards cancerous cells. In fact, HDFn cells survived at the highest tested treatment of 100 μg/mL of IVL DCM, a concentration that substantially reduced the viability of A549 cells (Figure 3A, lower panel).

The MTT assay is essentially a metabolic assay that indirectly measures cell viability; therefore, cell viability was evaluated using DAPI and crystal violet staining methods, which directly assess cell viability. A549 cells treated with varying concentrations of IVL DCM were stained with DAPI nuclear stain (Figure 3B) and crystal violet (Figure 3C), which labels adherent cells. Both crystal violet and DAPI staining confirmed that IVL DCM potently reduced the viability of A549 lung cancer cells in a concentration-dependent manner (Figure 3B,C).

### 3.4. Antioxidant Capacity and Total Phenolic and Flavonoid Contents of Dichloromethane Fraction of Inula viscosa Leaves Methanolic Extract (IVL DCM)

**Figure 4:**
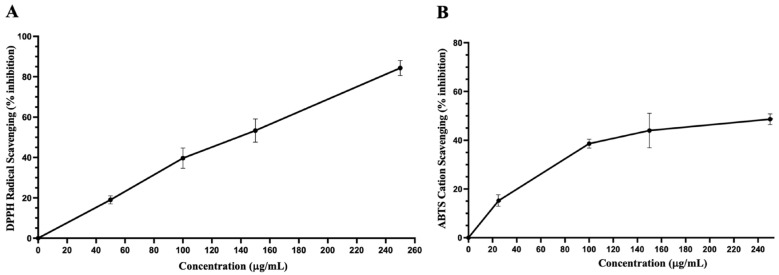
Antioxidant capacities of IVL DCM. (**A**) DPPH free radical scavenging activity of IVL DCM fraction (25–250 μg/mL). (**B**) ABTS• cation scavenging activity of IVL DCM fraction (25–250 μg/mL). Ascorbic acid was used as a reference. Values are expressed as means ± SEM (*n* = 3). These graphs were used to calculate EC_50,_ as shown in Table 2.

Table 2 shows TPC, TFC, and the antioxidant capacity of IVL DCM fraction. IVL DCM showed high TPC and TFC contents and a robust antioxidant capacity similar to that of IVLM (Figure 4 and Table 4).

### 3.5. Gas Chromatography-Mass Spectroscopy (GC-MS) of Dichloromethane Fraction of Inula viscosa Leaves Methanolic Extract (IVL DCM)

**Figure 5:**
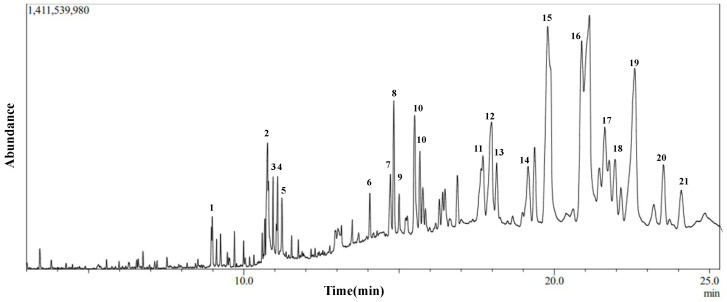
Chromatogram of GC-MS analysis of IVL DCM fraction representing the elution profile of metabolites listed in Table 5.

To elucidate the chemical composition of the IVL DCM fraction and determine the cause of its cytotoxic and antioxidant properties, GC-MS analysis was performed. Figure 5 shows the GC chromatogram of IVL DCM. Table 5 lists 21 of the major metabolites (phytochemicals) of IVL DCM identified by comparing their mass spectral fragmentation patterns to known compounds in the NIST library. Indeed, most of the metabolites identified in the IVL DCM fraction are terpenoids, indicating the enrichment of terpenoids in this fraction. Among the 21 identified metabolites, 11 were terpenoids, which included monoterpenes (isopulegol, linalyl propionate, and citronellal), oxygenated sesquiterpenes (caryophyllene oxide), and triterpenoids (betulin, 9,19-cyclolanostan-3-ol acetate, and lupeol and its derivatives) (Table 5). The identified terpenoids could contribute to the medicinal qualities of the plant. Notably, some of the metabolites, such as ammodendrine, isopulegol, lupeol and its derivatives, betulin, phytonadione, norcodeine, and hexadecyl oxirane, are described in *I. viscosa* for the first time (Table 5).

### 3.6. Inula viscosa Leaves Terpenoid-Rich Fraction (IVL DCM) Inhibited the Proliferation of A549 Lung Cancer Cells

**Figure 6:**
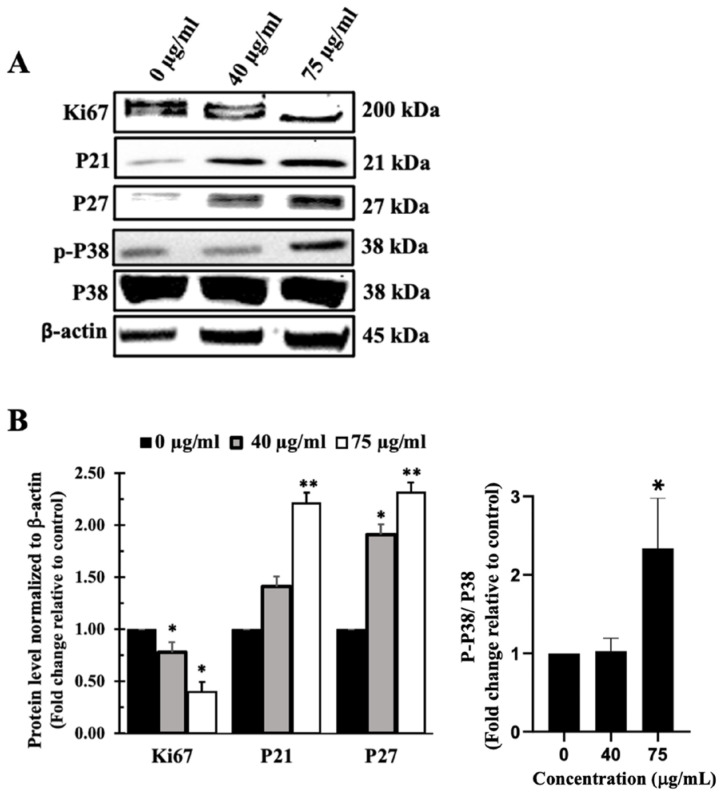
IVL DCM fraction inhibited the proliferation of A549 cells. A549 cells were treated with the indicated concentrations of IVL DCM for 48 h. (**A**) Ki67, P21, P27, p-P38, and P38 protein levels as detected by immunoblotting of A549 cell lysates. (**B**) Quantification of the bands in (**A**). Bar graphs of band intensity of target proteins normalized to the intensity of the loading control β-actin expressed as fold change of the vehicle-control and represented as the mean ± SEM of three independent experiments (*n* = 3). p-P38 protein levels were normalized to P38 protein. * *p* < 0.05 and ** *p* < 0.01.

Western blotting of the cell proliferation markers Ki67, P21, and P27 confirmed that IVL DCM-mediated reduction of cell viability takes place at least partly through inhibition of cellular proliferation of A459 cells (Figure 6). Treatment of A549 cells with IVL DCM significantly (*p* < 0.05) reduced Ki67 protein levels in a concentration-dependent manner. IVL DCM concentrations of 40 μg/mL and 75 μg/mL caused 0.78 ± 0.05 and 0.40 ± 0.05-fold reduction in Ki67 protein levels.

Figure 6 also shows that treatment of A549 cells with IVL DCM activated the mitogen-activated protein kinase (MAPK) P38, an essential regulator of cell proliferation [66]. Treatments of A549 cells with 75 μg/mL IVL DCM significantly (*p* < 0.05) increased the phosphorylation of P38 at Thr180/Tyr182 by 2.33 ± 0.70 folds (Figure 6B). Consistently, activation of P38 manifested in elevated levels of downstream effectors, specifically CDK inhibitor proteins P21 and P27, as depicted in Figure 6. IVL DCM concentrations of 40 and 75 μg/mL increased P21 protein levels at the treatment concentrations of 40 and 75 μg/mL of IVL DCM (1.41 ± 0.01 and 2.21 ± 0.05-fold change, respectively), but the increase was significant (*p* < 0.01) only at 75 μg/mL of IVL DCM. P27 protein levels also increased significantly at the treatment concentrations of 40 μg/mL (*p* < 0.05) and 75 μg/mL (*p* < 0.01) of IVL DCM (1.91 ± 0.6 and 2.31 ± 0.35-fold change, respectively).

### 3.7. I. viscosa Leaves Terpenoid-Rich Fraction (IVL DCM) Induced Apoptosis of A549 Cells

**Figure 7:**
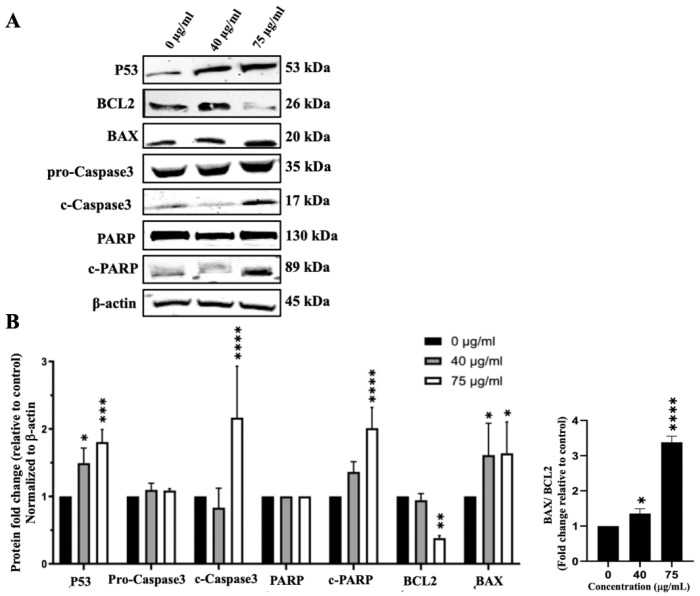
IVL DCM induces the apoptosis of A549 cells. A549 cells were treated with the indicated concentrations of IVL DCM for 48 h. (**A**) P53, BCL2, BAX, Caspase 3, c-Caspase 3, PARP, c-PARP protein levels as detected by immunoblotting of A549 cell lysates. β-actin was immunoblotted as a loading control (**B**). Quantification of the bands in (**A**). Bar graphs of band intensity of target proteins normalized to the intensity of the loading control β-actin expressed as fold change of the vehicle control and represented as the mean ± SEM of three independent experiments (*n* = 3). The right panel of (**B**) shows the ratio of BAX/BCL2 (*n* = 3). * *p* < 0.05, ** *p* < 0.01, *** *p* < 0.001, and **** *p* < 0.0001.

In addition to inhibiting cell proliferation, IVL DCM could reduce cell viability by inducing apoptosis. To detect the effects of IVL DCM fraction on the apoptosis pathway, protein levels of Caspase3, c-Caspase3, BCL2, BAX, PARP, c-PARP, and P53 were evaluated by immunoblotting of A549 cells treated with 40 and 75 μg/mL of IVL DCM. P53 protein levels gradually and significantly increased when increasing the concentration of IVL DCM from 40 (*p* < 0.05) to 75 μg/mL (*p* < 0.001) by 1.49 ± 0.13 and 1.81 ± 0.10 folds, respectively (Figure 7), indicating the activation of the apoptotic pathway. Protein levels of activated apoptosis effector enzyme c-Caspase3 were significantly (*p* < 0.0001) elevated by 75 μg/mL of IVL DCM (2.17 ± 0.44-fold change) (Figure 7). Similar to P53, the protein levels of the active PARP enzyme, c-PARP, increased dose-dependently at 40 and 75 μg/mL of IVL DCM. However, the increase was significant (*p* < 0.0001) only at 75 μg/mL of IVL DCM (2.01 ± 0.16-fold change) (Figure 7). The levels of the anti-apoptotic protein BCL2 decreased significantly (*p* < 0.0001) by 0.38± 0.05 folds at 75 μg/mL of IVL DCM (Figure 7). In contrast, the levels of the proapoptotic protein BAX increased significantly (*p* < 0.001) by 1.61 ± 0.33 and 1.64 ± 0.34 folds at 40 and 75 μg/mL of IVL DCM, respectively (Figure 7). The ratio of BAX/BCL2, which is indicative of induction of apoptosis, increased significantly (*p* < 0.001) at 40 and 75 μg/mL of IVL DCM by 2.26 ± 0.34 and 5.25 ± 1.26 folds, respectively.

These results indicate that IVL DCM-triggered reduction in cell viability was mediated at least partly by the intrinsic apoptotic pathway.

### 3.8. Inula viscosa Leaves Terpenoid Enriched Fraction (IVL DCM) Reduced the Migration of A549 Lung Cancer Cells through Reduction of FAK Activation

**Figure 8:**
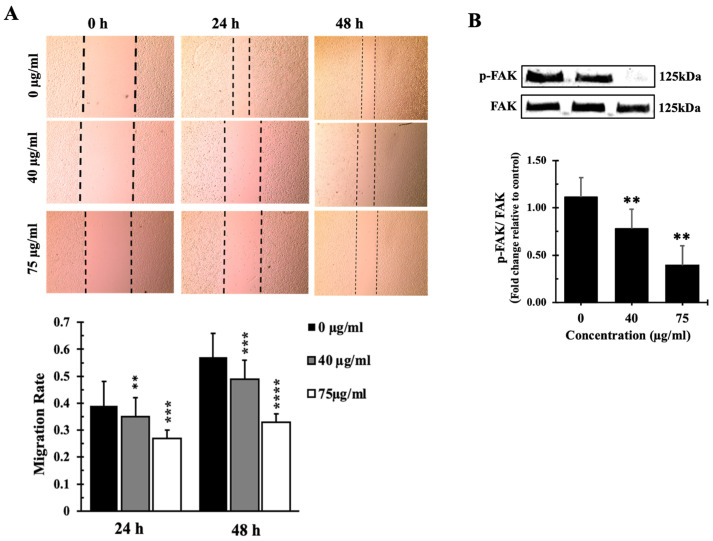
IVL DCM fraction inhibited the migration of A549 cells. (**A**) a confluent monolayer of A549 cells was wounded by scratching. The cells were then incubated with the indicated concentrations of IVL DCM fraction. The wound was imaged 24 and 48 h after treatment, and the images were analyzed to quantify cell migration using Image J software. Values represent the fold change in migration compared to the vehicle-untreated cells. Bar graphs in the lower panel in (**A**) represent the migration rate of the cells after 24 and 48 h of treatment. (**B**) Western blotting using anti-pFAK and anti-FAK antibodies. Values represent the average of three independent experiments (*n* = 3) and are expressed as mean ± SEM. ** *p* < 0.01, *** *p* < 0.001, and **** *p* < 0.0001.

The impact of the IVL DCM terpenoid-rich fraction on A549 cell migration was assessed using a wound healing/scratch assay. Results of the wound healing assays revealed a remarkable reduction in cell migration at the concentrations of 40 and 75 µg/mL, demonstrating a 65% and 73% decrease in cell migration after 24 h, respectively, and a 51% and 67% decrease in cell migration after 48 h, respectively (Figure 8A). This suggests that IVL DCM has a considerable inhibitory effect on A549 cell migration, highlighting its potential antimetastatic properties.

IVL DCM-induced reduction in cell migration appears to be FAK-mediated. Treatment of A549 cells with 40 and 75 µg/mL of IVL DCM for 48 h reduced the activation of FAK (Figure 8B), which plays a crucial role in cellular adhesion and migration, further supporting the antimigratory effects of IVL DCM. Treatment with 40 μg/mL and 75 μg/mL IVL DCM reduced the phosphorylation of FAK at Tyrosine 397 by 0.75 ± 0.21 and 0.35 ± 0.18 folds, respectively, underscoring the involvement of FAK in IVL DCM-induced inhibition of migration.

## 4. Discussion

### 4.1. Phenolic and Flavonoid Contents and Antioxidant Capacity of I. viscosa Parts

In this study, the different extraction solvents (methanol vs. water) demonstrated a significant variation in the extraction yield, total phenolic (TPC) and flavonoid contents (TFC), as well as antioxidant capacity. Study findings revealed that the methanolic solvent had higher extraction yields for both *I. viscosa* plant parts under study (i.e., leaves and stems) than the water solvent. Of note, the extraction yields, TPC, and TFC of the *I. viscosa* aqueous extracts in this study are lower than those reported by other studies [34]; this could be due to differences in extraction and storage conditions as well as the time of collection and geographical location of the plant material. Methanolic extracts also had greater TPC and TFC than their aqueous counterparts. These findings are in alignment with previous studies on *I. viscosa* methanolic and aqueous extracts [34,67,68]. Relatedly, the high polarity of the water and methanol solvents is considered essential in improving TPC and the antioxidant capacity of corresponding plant extracts [69,70,71].

Furthermore, *I.* viscosa leaves had higher extraction yield, TPC, and TFC than stems, irrespective of the solvent used, indicating the richness of leaves in phenolic and flavonoid secondary metabolites. This is also in agreement with the literature, where leaves frequently have a higher TPC and TFC than stems [72,73,74,75,76]. The higher TPC and TFC in leaves than stems may be related to the abundance of epidermal tissue, mesophyll, parenchyma, and secretory glands, which usually store secondary metabolites in plant leaves rather than stems, which are mainly made of transport tissues, such as the xylem and phloem [77]. In addition, higher TPC is also correlated with higher activity of phenylalanine ammonia-lyase (PAL), a major enzyme in the biosynthesis of phenolic acids. PAL activity levels vary in different parts of a plant depending on harvest time, light intensity, and geographical location, among other factors [73]. Future studies should investigate PAL activity between *I. viscosa* leaves and stems. On the other hand, plants may have higher TPC or TFC in stems rather than leaves because the concentration and distribution of phytochemicals in plant parts are not only influenced by genetics but are also affected by many environmental factors, including light, humidity, and soil [72].

Similarly, the antioxidant activity varied substantially depending on the extraction solvent and plant part. The observed higher DPPH and ABTS antioxidant capacity of leaves extracts in both solvents (IVLM and IVLaq), as compared to those of the stems, can be related to differences in the chemical composition of these plant parts and the chemical properties of the phytochemical compounds potentially responsible for the antioxidant activity, such as phenolic and flavonoid compounds. These antioxidant phytochemicals could have a higher solubility in methanol than water, hence their higher antioxidant capacity in the methanol extract [11,69,70,71,78,79,80]. Moreover, the higher yields and antioxidant activity in the methanol solvent could be related to its polar constituents [81].

Plant phytochemicals exhibit their antioxidant activity through different mechanisms of action associated with their structural specificities [82]. Phenolic and flavonoid antioxidant phytochemicals scavenge reactive species of oxygen, nitrogen, and chlorine and chelate metal ions at both the initiation step and during the progress of the oxidative process [82]. Our findings indicated that the antioxidant capacity of *I. viscosa* extracts generally correlated with higher TFC and TPC contents, aligning with the literature underscoring that phenolic and flavonoid metabolites are the main contributors to a plant extract’s antioxidant capacity [82]. Interestingly, the DPPH and ABTS antioxidant capacity positively correlated with TFC and TPC in the case of *I. viscosa* stems but not leaves, despite the solvent used. This may suggest a difference in the chemical nature and solubility of compounds responsible for the antioxidant properties between the stems and leaves [82]. In addition, similar to the TFC and TPC, *I. viscosa* leaves had higher antioxidant activity than stems, which is in line with reports in the literature [72,73,74,75,76]. The high phenolic content and antioxidant properties of *Inula viscosa* in this study are substantiated by previous studies [31,83,84,85], highlighting the relevance of the capacity of a plant extract to modulate oxidative stress with its therapeutic properties, including anticancer effects [83].

### 4.2. Inula viscosa Leaves Extracts Reduced the Viability of A549 Lung Cancer Cells

In this study, *I. viscosa* leaves and *stems* methanolic extract demonstrated a significant reduction in cancer cell viability, confirming the findings of previous studies illustrating the cytotoxic potential of *I. viscosa* extracts against various cancer cell lines [21,24,25,34]. For instance, the recent research by Kheyar et al. (2022) [25] revealed a high effectiveness of *I. viscosa* leaves ethanolic extract in reducing the proliferation of a human colorectal adenocarcinoma cell line (HT29) with an IC50 value of 62.39 ± 0.34 μg/mL.

Similar to this study, Rechek et al. [30] reported that *I. viscosa* reduced the viability of A549 lung cancer cells. However, Rechek et al. tested *I.* viscosa leaves extracts [30], while we tested a methanolic extract of *I. viscosa* leaves. Moreover, we found that *I. viscosa* leaves methanolic extracts reduced the viability of human breast, ovary, colorectal, and liver cancer cells, which is also consistent with previous reports [15,26,28].

Unlike the *Inula viscosa* methanolic extracts, the aqueous extracts of leaves and stems did not reduce the viability of A549 lung cancer cells in this study. These findings are in agreement with the findings of Anglana et al., who found that the aqueous extracts of *I. viscosa* aerial parts have variable cytotoxicity against different colorectal cancer cells, being moderately cytotoxic to SW620 cells and noncytotoxic to DLD-1 and HT-29 cells. In contrast, the methanolic extracts, prepared under the same conditions, were overall more cytotoxic to all the tested colorectal cancer cell lines [26], and the results by Kheyar-Kraouche et al., who showed that *I. viscosa* ethanolic extracts reduced the viability of HepG2 liver cells more efficiently than the aqueous extract [34]. Likewise, Colak et al. showed that *I. viscosa* methanolic extracts were cytotoxic to several cancer cells, while I. viscosa aqueous extracts did not cause significant cell death in most of the tested cell lines [28]. This contrasts the results of Hepokur et al., who reported that *I. viscosa* aqueous extracts are cytotoxic to several cancer cell lines [86]. However, Hepokur et al. prepared the aqueous extract by boiling *I. viscosa* powder at 100 °C while we prepared the aqueous extract by maceration at room temperature.

The variation in the cytotoxic effects between methanolic vs. aqueous extracts could be related to the higher extraction yield and, subsequently, higher TPC, TFC, and antioxidant capacity of the methanol solvent vs. water solvent. In confirmation, many studies have demonstrated that the TPC, TFC, and antioxidant capacity of a plant extract are related to its therapeutic effects, including antiproliferative and anticancer properties [11,66,87,88,89,90,91]. In this study, the high TPC and TFC of *I. viscosa* leaves methanolic extracts may underline their cytotoxic and antiproliferative effects, especially since many polyphenols and flavonoids, acting through diverse metabolic and signaling pathways, have been shown to cause significant inhibition of the proliferation of various types of cancer cells [87,90,92,93].

### 4.3. A Terpenoid-Rich Fraction of I. viscosa (IVL DCM)

Given the scarcity of reports on the anti-lung cancer effects of *I. viscosa* extracts in general and *I. viscosa* terpenoids in particular, we further purified *I. viscosa* leaves methanolic extracts (IVLM) in DCM to enrich *I. viscosa* terpenoids, obtaining the IVL DCM fraction. GC-MS analysis confirmed the abundance of terpenoids in the IVL DCM fraction. The enrichment of terpenoids in this fraction is consistent with other studies that used a low-to-intermediate-polarity solvent to recover slightly polar terpenoids [38,45].

### 4.4. The Terpenoid-Rich Fraction of I. viscosa Reduced the Proliferation and Migration and Induced Apoptosis of A549 Lung Cancer Cells

The IVL DCM fraction reduced the viability of A549 and other cancerous cells. Both the terpenoid and non-terpenoid metabolites identified in the IVL DCM fraction corroborate the cytotoxic effects of IVL DCM. Terpenoids have diverse biological activities, including anticancer properties, and have been shown to significantly inhibit the proliferation of A549 and other cancerous cells [41,42,43,44]. Among the identified terpenoids, lupeol and its triterpene derivatives are highly represented in the IVL DCM fraction and have been demonstrated to have anticancer activities in cells in vitro and in vivo in mouse models [94,95,96]. Betulin, another triterpenoid present in the IVL DCM fraction, was reported to have anticancer effects [97,98]. Caryophyllene oxide is an oxygenated sesquiterpene that has been documented to have important anticancer activities, impacting the growth and proliferation of various cancer cells [99,100,101,102,103]. Isopulegol is a monoterpene that has exhibited in vitro cytotoxic effects against several cancer cell lines [104,105]. In addition, some of the non-terpenoid metabolites have documented anticancer effects and could contribute to the anticancerous activity of the IVL DCM fraction, δ-tocopherol [106,107] and phytonadione [108,109], for instance.

The terpenoid-rich fraction reduced the viability of A549 cells, at least partly by inhibiting the proliferation of A549 cells. IVL DCM reduced the protein levels of the proliferation marker Ki67 and elevated the protein levels of p-P38 and its downstream targets CDK inhibitor proteins P21 and P27. These results align with previous studies reporting that P38 activation slows down the proliferation and increases the expression levels of P21 in lung cancer cells [110].

The results of examining the apoptotic activity of the terpenoid-rich fraction contribute insights into the mechanisms underlying the anticancer properties of *I. viscosa*. The terpenoid-rich fraction activated the intrinsic apoptotic pathway. Indeed, IVL DCM elevated the levels of the P53 and proapoptotic protein BAX, reduced the levels of the anti-apoptotic protein BCL2, and activated the effector apoptosis enzyme Caspase 3 into c-Caspase 3, which activated PARP into c-PARP. In addition, the ratio of BAX/BCL2 was increased, further confirming the induction of apoptosis through the intrinsic apoptotic pathway. It is well established that various signals induce the expression of P53, which activates several transcriptional programs that induce cell cycle arrest [8], DNA repair [8], senescence, or apoptosis, leading to the suppression of tumor growth [111]. P53 activation can trigger the intrinsic apoptotic pathway, leading to the release of cytochrome c from mitochondria, activation of Caspase 9 and Caspase 3, and reduction and induction of BCL2 and BAX protein levels, respectively [112,113,114]. Active Caspase 3 is an apoptosis effector enzyme that cleaves various protein substrates, such as caspase-activated DNAse that fragments genomic DNA and PARP-1, to induce apoptosis [11,114]. The results are in agreement with previous studies showing that I. viscosa extracts can induce apoptosis in cancerous cells [24] and in A549 cells [30]. Rechek et al. [30] reported that *I. viscosa* induced apoptosis in A549 lung cancer cells, similar to this study. However, Rechek et al. reported that *I. viscosa* leaves ethanolic extracts induced apoptosis through activation of RIPK1, unlike this study where IVL DCM activated the intrinsic apoptotic pathway [30]. This could be due to the difference in the chemical composition of the ethanolic leaves extract of *I. viscosa* leaves prepared by Rechek et al. and the terpenoid-rich fraction prepared in this study.

Furthermore, the results of this study are consistent with studies demonstrating that terpenoids can significantly inhibit proliferation and induce apoptosis in A549 and other cancerous cells [41,42,43,44]. For example, *I. viscosa* sesquiterpene lactones can inhibit the growth and metastasis of human cancer cells, induce apoptosis, autophagy, and cell cycle arrest, and increase the sensitivity of chemotherapy drugs, activate the p38 MAPK pathway, and inhibit the NF-κB pathway in lung cancer [115]. In addition, several terpenoids that we identified in the IVL DCM fraction are known to induce apoptosis [94,95,96,97,98,99,100,101,102,103,104,105]. For instance, caryophyllene oxide has been shown to induce apoptosis of A549 cells [99] and has been reported to induce apoptosis through the PI3K/AKT/mTOR/S6K1 pathway [101]. Phytonadione (Vitamin K1) is a non-terpenoid metabolite in IVL DCM, which induces apoptosis in colon cancer cell lines [108].

Cell migration is required for efficient tumor metastasis as cells spread away from the primary tumor site [116,117]. In this study, a wound healing assay revealed that IVL DCM inhibited the. migration of A549 cells. In accordance, *I. viscosa* methanol extracts were shown to suppress migration and induce cytotoxicity and apoptosis of melanoma cell lines [28]. Furthermore, terpenoids have been reported to inhibit cancer cell migration [118]. In contrast, other studies have reported that *I. viscosa* extracts can enhance migration of fibroblast cells, suggesting that the extracts may be beneficial as wound healing agents [119]. The difference in effects on cell migration may be related to the difference in chemical composition between the terpenoid-rich IVL DCM fraction and the *I. viscosa* extracts with wound healing activity and the kind of cells used (cancerous human cells vs. mouse fibroblasts). For example, tomentosin, a sesquiterpene lactone highly abundant in *I. viscosa*, was shown to inhibit migration of osteosrcoma [120] and multiple meyeloma cells [121] and downregulate genes enriched in migration, proliferation, growth, and invasion pathways [121]. Andrographis is a diterpene that was shown to inhibit migration of A549 cells [122].

Focal adhesion kinase (FAK) promotes the migration and invasion of tumor cells [123,124,125,126]. FAK levels are frequently high in cancerous cells. High FAK levels correlate with human cancers’ metastatic potential [127,128,129]. IVL DCM-mediated inhibition of cell migration was accompanied by inhibition of FAK activation. The latter event may underpin the attenuated migratory potential of A549 cells. This is the first report of the correlation between inhibition of cell migration and suppression of FAK activation by *I. viscosa* extracts. Interestingly, terpenoids have been shown to inhibit FAK activation. Isomalabaricane triterpenoids have been shown to target FAK [130]. Oridonin diterpenoid inhibits the FAK signaling pathway in human small cell lung cancer cells H1688 [131]. However, it remains to be tested if the inhibition of FAK activation is the only mechanism through which IVL DCM inhibits A549 cell migration or if IVL DCM may target other cell migratory pathways.

This study has given significant insights into the mechanisms of action underlying the anticancer effects of *I. viscosa* bioactivities. Uncovering the full therapeutic potential of *I. viscosa* remains a daunting task due to the complexity and variability of *I. viscosa* extracts, which contain hundreds or even thousands of individual bioactive compounds of varying abundance and identifying compounds responsible for a given biological activity is by itself a very ambitious endeavor. This task is further complicated by the fact that the overall activity of extracts of medicinal plants is possibly a result of the combined action of multiple compounds with synergistic, additive, or antagonistic activity [132].

In conclusion, this study revealed the cytotoxic effects of a methanolic extract of *I. viscosa.* Furthermore, a terpenoid-rich fraction was prepared and characterized by GC-MS. The terpenoid-rich fraction is rich in several terpenoids, identified for the first time in I. viscosa, that can explain its ability to inhibit proliferation and migration and induce the intrinsic apoptotic pathway in A549 cells. These results warrant future purification of the *I. viscosa* terpenoid-rich fraction to isolate its metabolites responsible for the observed anticancer effects. Overall, our study revealed the potential anti-cancerous effects of *I. viscosa* terpenoids against lung cancer cells, affirming *I. viscosa* as a renewed source for the discovery of potential anticancer drugs.

## Figures and Tables

**Table 1:** Extraction yield, total phenolic content (TPC), total flavonoid content (TFC), and antioxidant capacity of *I. viscosa* leaves and stems methanolic or aqueous extracts. DPPH and ABTS EC_50_ of L-ascorbic acid are shown for comparison. IVLM: *I. viscosa* leaves methanolic extract; IVSM: *I. viscosa* stems methanolic extract; IVLaq: *I. viscosa* leaves aqueous extract; IVSaq: *I. viscosa* stems aqueous extract.

Plant Extract	Extraction Yield (%)	TPC (µg GAE/g)	TFC (µg QE/g)	DPPH EC_50_ (µg/mL)	ABTS EC_50_ (µg/mL)
IVLM	16.5	726.4 ± 1.1	303.3 ± 8.8	145.7 ± 2.6	236.9 ± 22.2
IVSM	4.4	532.0 ± 10.3	114.0 ± 4.4	229.7 ± 3.1	239.0 ± 5.5
IVLaq	3.5	212.0 ± 1.5	79.8 ± 2.9	155.8 ± 6.1	268.9 ± 3.7
IVSaq	2	174.3 ± 0.7	45.2 ± 0.1	693.8 ± 3.4	791.0 ± 14.5
L-Ascorbic acid	─	─	─	27.5 ± 1.3	93.4 ± 0.9

**Table 2:** IC_50_ values of IVLM-induced reduction in viability of SK-OV-3, MCF-7, HepG2, and HCT116 human cancer cell lines at 24 h. MTT assay was performed as described for A549 cells.

Cell Line	IC_50_ (μg/mL)
SK-OV-3	80.0 ± 5.7
MCF-7	54.0 ± 4.9
HepG2	59.9 ± 7.5
HCT116	39.2 ± 6.1

**Table 3:** IC_50_ values of IVL DCM-induced reduction in viability of SK-OV-3, MCF-7, MDA-MB-231, HepG2, and HCT116 human cancer cell lines at 24, 48, and 72 h. MTT assay was performed as described for A549 cells. NT: not tested.

IC_50_ (μg/mL)			
Cell Line	24 h	48 h	72 h
SK-OV-3	110.2 ± 6.9	96.5 ± 4.0	52.95 ± 6.7
MCF-7	84.4 ± 6.1	42.5 ± 2.9	29.32 ± 1.2
MDA-MB-231	86.8 ± 4.8	69.3 ± 3.3	49.09 ± 1.8
HepG2	NT	20.2 ± 5.2	NT
HCT116	NT	19.7 ± 3.7	NT

**Table 4:** Total phenolic content (TPC), total flavonoid content (TFC), and antioxidant capacity of *I. viscosa* leaves methanolic crude extract extracted with dichloromethane (IVL DCM).

	*TPC* (µg GAE/g)	*TFC* (µg QE/g)	*DPPH EC_50_* (µg/mL)	*ABTS EC_50_* (µg/mL)
IVL DCM	724.4 ± 12.1	235.4 ± 5.1	143.0 ± 1.4	241.6 ± 9.7

**Table 5:** GC-MS analysis of IVL DCM fraction. The identified metabolites’ names, chemical nature, retention time (RT), molecular formula, and weight are presented. The listed phytochemicals were identified using the NIST database. The last column shows reference publications that previously identified these phytochemicals in *I. viscosa.* NR: not reported in *I. viscosa* before.

No.	Compound Name	Chemical Nature	RT (min)	Molecular Formula	Molecular Weight	Reference
1	2-Hexyldecan-1-ol	Alcohol	8.995	C_16_H_34_O	242	NR
2	Pyridine, 1-acetyl-1,2,3,4-tetrahydro-5-(2-piperidinyl)-(Ammodendrine)	Pyridine alkaloid	10.77	C_12_H_20_N_2_O	208	NR
3	Isopulegol	Monoterpene	10.95	C_10_H_18_O	154	NR
4	Linoleic acid ethyl ester	Fatty acid derivative	11.06	C_20_H_36_O_2_	308	[51,52,53]
5	Caryophyllene oxide	Oxygenated sesquiterpene	11.10	C_15_H_24_O	220	[53,54,55,56,57,58,59,60,61,62]
6	3,25-bis(acetyloxy)-5-hydroxyergostan-6-one	Steroid	13.90	C_32_H_52_O_6_	532	NR
7	Citronellal	Monoterpene	14.60	C_10_H_18_O	154	[63]
8	Lup-20(29)-en-3-one (Lupenone)	Triterpenoid	14.85	C_30_H_48_O	424	NR
9	δ-Tocopherol	Vitamin E	15.23	C_27_H_46_O_2_	402	[64,65]
10	Lupeol, trifluoroacetate	Triterpene	15.69	C_32_H_49_F_3_O_2_	522	NR
11	Linalyl propionate	Monoterpene	17.73	C_13_H_22_O_2_	210	[53,60]
12	Betulin	Triterpenoid	18.01	C_30_H_50_O_2_	442	NR
13	Phytyl palmitate	Fatty acid/diterpene derivative	18.20	C_36_H_70_O_2_	534	NR
14	Campesterol	Phytosterol	19.41	C_28_H_48_O	400	[65]
15	6-Octadecenoic acid derivative	Fatty acid derivative	19.83	C_22_H_41_NO	335	NR
16	Norcodeine	Alkaloid	21.10	C_17_H_19_NO_3_	285	NR
17	Phytonadione (Phylloquinone)	Vitamin K	21.50	C_31_H_46_O_2_	450	NR
18	Lup-20(29)-en-3beta-ol, acetate (20(29)-(Lupenol acetate)	Triterpenoid	21.67	C_32_H_52_O_2_	468	NR
19	Lupeol	Triterpenoid	22.63	C_30_H_50_O	426	NR
20	9,19-Cyclolanostan-3-ol acetate	Triterpenoid	23.77	C_32_H_54_O_2_	470	NR
21	2-Hexadecyloxirane	Oxirane	24.16	C_18_H_36_O	268	NR

## Data Availability

All data are available upon request from the corresponding author.

## Supplementary Materials

The following supporting information can be downloaded at: https://www.mdpi.com/article/10.3390/biology13090687/s1, Original Westen Blot figures with quantification.
