# Supplementary material for: Chemical Composition, Antioxidant Capacity, and Anticancerous Effects against Human Lung Cancer Cells of a Terpenoid-Rich Fraction of Inula viscosa
Source: Biology (Basel). 2024 Sep 2;13(9):687. doi: 10.3390/biology13090687 (PMC11429348; doi:10.3390/biology13090687)
Supplement: Supplementary file 1 [file biology-13-00687-s001.zip › biology-3118513-supplementary.pdf]

# FAK and p-FAK

## FAK

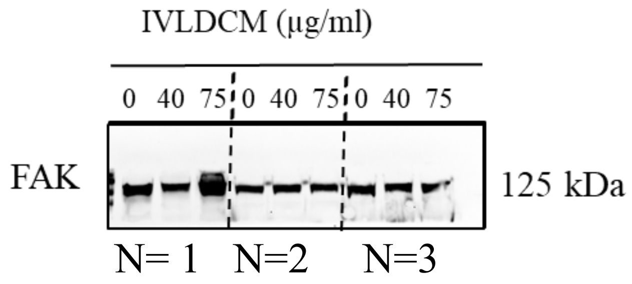


## p-FAK

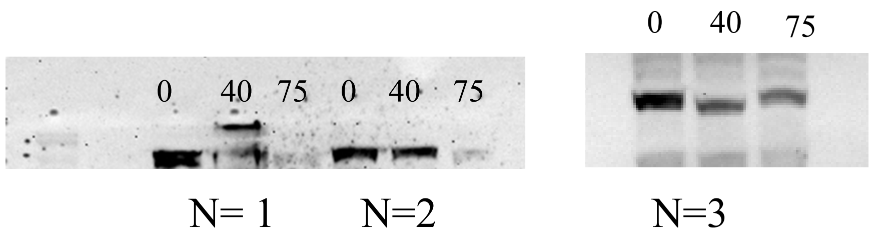


| FAK | A1 | A2 | A3 | |
|---|---|---|---|---|
| 0 ug/mL | 336 | 261 | 325 | |
| 40 | 384 | 352 | 310 | |
| 75 | 616 | 372 | 132 | |
| p-FAK | Area 1 | Area 2 | Area 3 | |
| 0 ug/mL | 348 | 330 | 336 | |
| 40 | 200 | 350 | 260 | |
| 75 | 50.00 | 72.00 | 101.00 | |
| p-Fak/Fak | fold change1 | fold change2 | fold change3 | Av fold change |
| 0 ug/mL | 1.04 | 1.26 | 1.03 | 1.11 |
| 40 | 0.52 | 0.99 | 0.84 | 0.78 |
| 75 | 0.08 | 0.19 | 0.77 | 0.35 |

## BAX

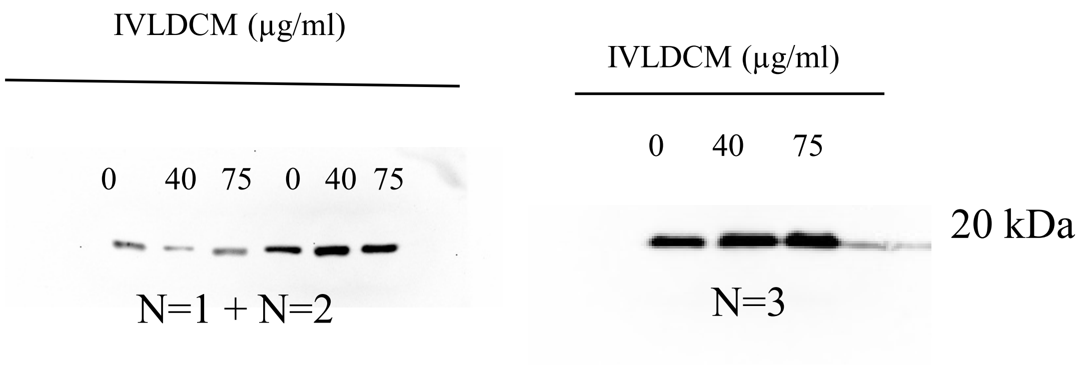


| BAX | A1 | A2 | A3 | Average A |
|---|---|---|---|---|
| 0 ug/mL | 180.00 | 250.00 | 380.00 | 270.00 |
| 40.00 | 145.00 | 400.00 | 450.00 | 331.67 |
| 75.00 | 240.00 | 400.00 | 490.00 | 376.67 |

## BCL2

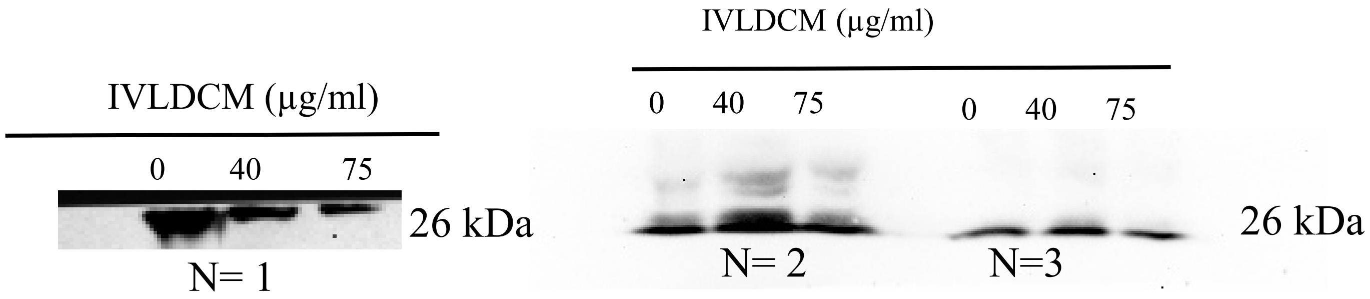


| BCL2 | A1 | A2 | A3 | average | BAX/BCL2 1 | BAX/BCL2 1 | BAX/BCL2 1 | Fold chande 1 | Fold change2 | Fold change 3 | average |
|---|---|---|---|---|---|---|---|---|---|---|---|
| 0 | 560 | 224 | 250 | 344.67 | 0.32 | 1.12 | 1.52 | 1.00 | 1.00 | 1.00 | 1.00 |
| 40 | 520 | 280 | 140 | 313.33 | 0.28 | 1.43 | 3.21 | 0.87 | 1.28 | 2.11 | 1.42 |
| 75 | 200 | 150 | 94 | 148.00 | 1.20 | 2.67 | 5.21 | 3.75 | 2.38 | 3.43 | 3.19 |

# p-P38

IVLDCM (µg/ml)

0 40 75 0 40 75

p-P38

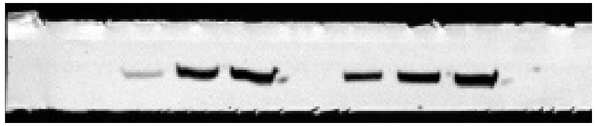

38 kDa

N= 1 N=2

IVLDCM (µg/ml)

0 40 75 0 40 75

p-P38

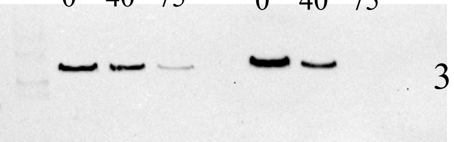

38 kDa

N=3

| p-P38 | area | area | area |
|---|---|---|---|
| 0 ug/mL | 105.00 | 92.00 | 78.00 |
| 40 | 114.00 | 150.00 | 96.00 |
| 75 | 189.00 | 260.00 | 234.00 |

IVLDCM (µg/ml)

0 40 75

P38

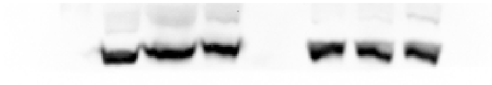

N= 1

0 40 75 0 40 75

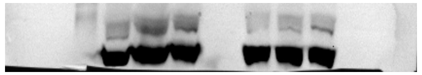

38 kDa

N= 2 N=3

| P38 | Area 1 | Area 2 | Area 3 | p-P38/P38 | p-P38/P38 | p-P38/P38 | Fold change 1 | Fold change 2 | Fold change 3 | Fold change |
|---|---|---|---|---|---|---|---|---|---|---|
| 0 ug/mL | 464 | 480 | 432 | 0.23 | 0.19 | 0.18 | 0.98 | 1.01 | 1.00 | 1.00 |
| 40 | 550 | 601 | 590 | 0.21 | 0.25 | 0.16 | 0.90 | 1.31 | 0.90 | 1.04 |
| 75 | 513 | 522 | 459 | 0.37 | 0.50 | 0.51 | 1.60 | 2.62 | 2.83 | 2.35 |

**PARP and c-PARP**

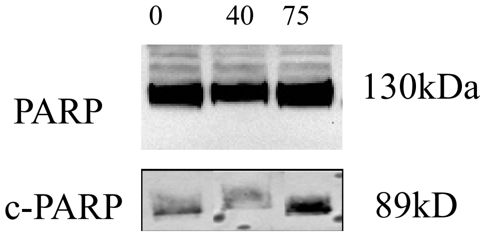


N=1

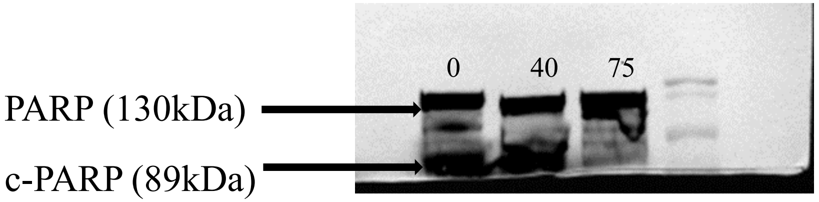


N=2

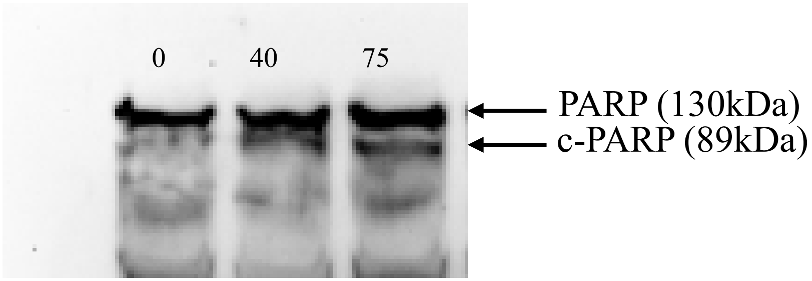


N=3

# PARP and c-PARP quantifications

| b-actin | Area1 | Area 2 | Area 3 |
|---|---|---|---|
| 0 ug/mL | 434 | 465 | 464 |
| 40.00 | 360 | 435 | 319 |
| 75.00 | 364 | 406 | 420 |

| PARP full lengh | Area 1 | Area 2 | Area 3 |
|---|---|---|---|
| 0 ug/mL | 330.00 | 374.00 | 396.00 |
| 40.00 | 352.00 | 396.00 | 374.00 |
| 75.00 | 420.00 | 432.00 | 363.00 |

| PARP/b-actin | PARP/B-actin | PARP/B-actin | PARP/B-actin | Fold change relative to control (0 ug/mL) | Fold change 1 | Fold change 2 | Average Fold change |
|---|---|---|---|---|---|---|---|
| 0 ug/mL | 0.76 | 0.80 | 0.85 | 1.00 | 1.01 | 1.00 | 1.00 |
| 40.00 | 0.98 | 0.91 | 1.17 | 1.29 | 1.14 | 1.38 | 1.27 |
| 75.00 | 1.15 | 1.06 | 0.86 | 1.52 | 1.33 | 1.02 | 1.29 |

| c-PARP | Area1 | Area 2 | Area 3 |
|---|---|---|---|
| 0 | 1156 | 600 | 1154 |
| 40 | 1732 | 1430 | 1000 |
| 75 | 1908 | 1800 | 1560 |

| c-PARP/b-actin | Area1 | Area 2 | Area 3 | Fold change 1 | Fold change 1 | Fold change 3 | Average Fold change |
|---|---|---|---|---|---|---|---|
| 0 | 2.66 | 1.29 | 2.49 | 1.00 | 1.00 | 1.00 | 1.00 |
| 40 | 4.81 | 3.29 | 3.13 | 1.81 | 2.55 | 1.26 | 1.87 |
| 75 | 5.24 | 4.43 | 3.71 | 1.97 | 3.44 | 1.49 | 2.30 |

# P53

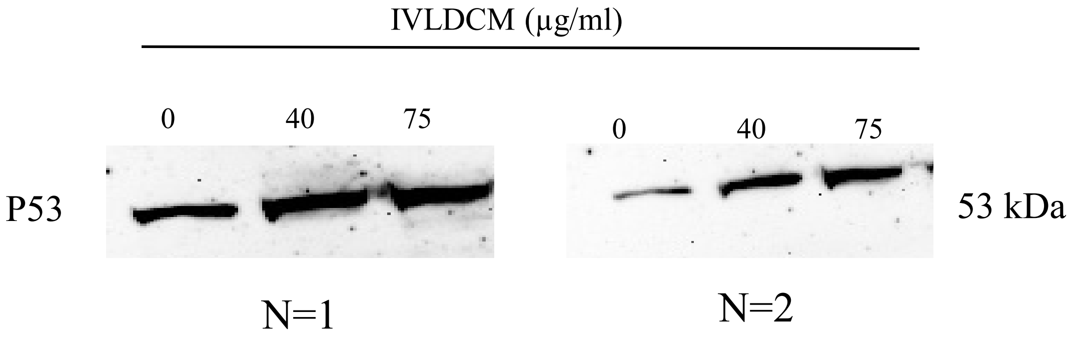


| p53 | A1 | A2 | b-actin | Area 1 | Area 2 | p53/b-actin 1 | p53/b-actin 2 | Fold change 1 | Fold change 2 | Average Fold change |
|---|---|---|---|---|---|---|---|---|---|---|
| 0 ug/mL | 350 | 116 | 0 ug/mL | 403 | 527 | 0.87 | 0.22 | 1.00 | 1.00 | 1.00 |
| 40 | 380 | 217 | 40 | 528 | 460 | 0.72 | 0.47 | 0.83 | 2.14 | 1.49 |
| 75 | 450 | 350 | 75 | 580 | 580 | 0.78 | 0.60 | 0.89 | 2.74 | 1.82 |

# Caspase 3

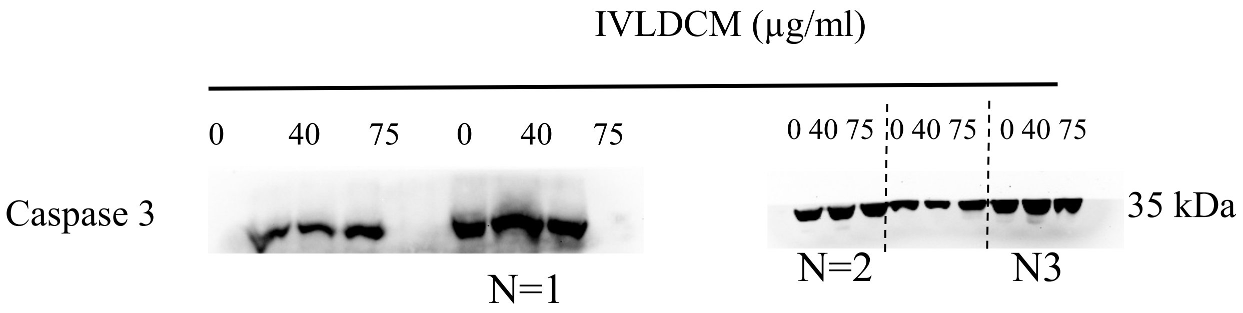


| b-actin | Area 1 | Area 2 | Area 3 | Caspase3 | Area 1 | Area 2 | Area 3 | C3/b-actin 1 | C3/b-actin 1 | C3/b-actin 1 | **Fold change 1** | **Fold change 2** | **Fold change 3** | **Average** |
|---|---|---|---|---|---|---|---|---|---|---|---|---|---|---|
| 0 ug/mL | 403 | 512 | 527 | 0 ug/mL | 464 | 480 | 432 | 1.15 | 0.94 | 0.82 | 1.00 | 1.00 | 1.00 | 1.00 |
| 40 | 528 | 476 | 434 | 40 | 476 | 504 | 522 | 0.90 | 1.06 | 1.20 | 0.78 | 1.13 | 1.47 | 1.13 |
| 75 | 484 | 511 | 442 | 75 | 513 | 522 | 459 | 1.06 | 1.02 | 1.04 | 0.92 | 1.09 | 1.27 | 1.09 |

# c-Caspase 3

IVLDCM (μg/ml)

0 40 75 0 40 75

c-Caspase3

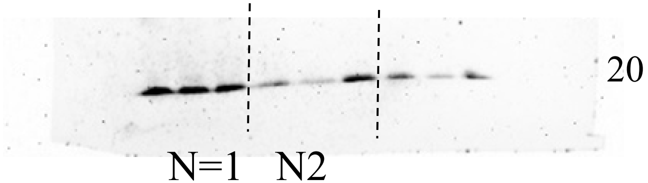

20 kDa

N=1 N2

IVLDCM (μg/ml)

0 40 75

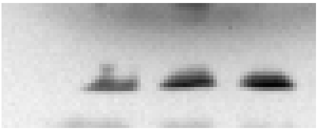

20 kDa

N=3

| c-Caspase3 | A1 | A2 | A3 | B-actin | A1 | A2 | A3 | CC3/b-actin 1 | CC3/b-actin 2 | CC3/b-actin 3 | Fold change 1 | Fold change 2 | Fold change 3 | fold change average |
|---|---|---|---|---|---|---|---|---|---|---|---|---|---|---|
| 0 ug/mL | 270 | 117 | 95 | 0 ug/mL | 300 | 200 | 182 | 0.90 | 0.59 | 0.52 | 1.00 | 0.99 | 1.00 | 1.00 |
| 40 | 300 | 130 | 119 | 40 | 216 | 196 | 234 | 1.39 | 0.66 | 0.51 | 1.54 | 1.12 | 0.98 | 1.22 |
| 75 | 400 | 300 | 320 | 75 | 270 | 260 | 297 | 1.48 | 1.15 | 1.08 | 1.65 | 1.96 | 2.07 | 1.89 |

# P21

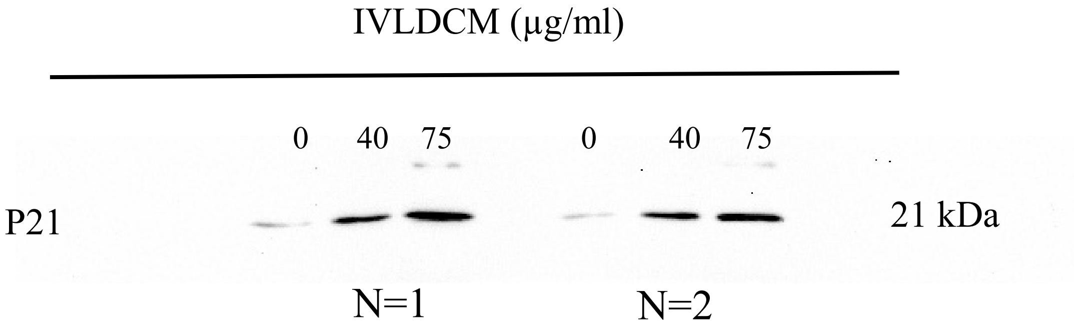


| p21 | Area 1 | Area 2 | b-actin | Area 1 | Area 2 | P21/b-actin | P21/b-actin | Fold change 1 | Fold change 2 | Fold change average |
|---|---|---|---|---|---|---|---|---|---|---|
| 0 | 175 | 150 | 0 | 243 | 200 | 0.72 | 0.75 | 1.00 | 1.00 | 1.00 |
| 40 | 230 | 201 | 40 | 216 | 196 | 1.06 | 1.03 | 1.48 | 1.37 | 1.42 |
| 75 | 450 | 410 | 75 | 270 | 260 | 1.67 | 1.58 | 2.31 | 2.10 | 2.21 |

# Ki67

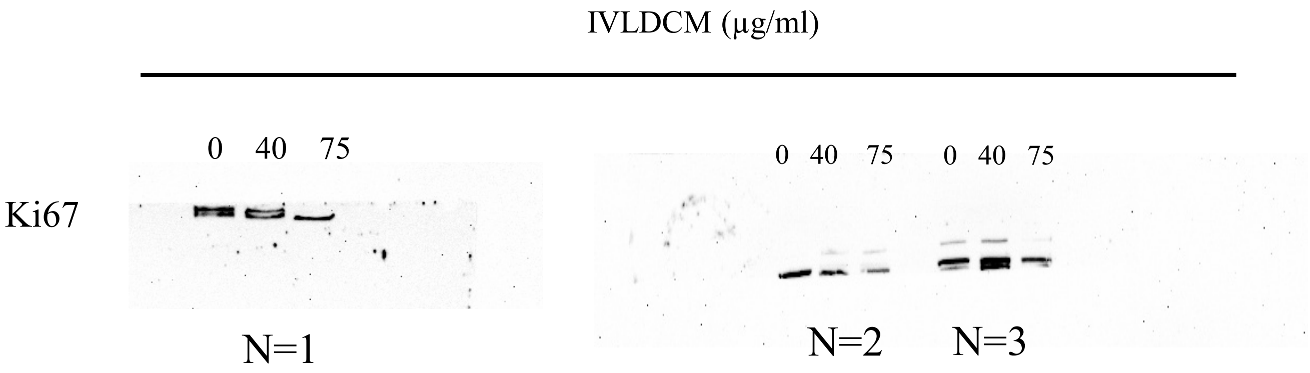


| b-actin | | | | | Ki67 | | | | | | | | | | |
|---|---|---|---|---|---|---|---|---|---|---|---|---|---|---|---|
| Concentration (µg/ml) | area | area | area | av | Concentration (µg/ml) | Area 1 | Area 2 | Area 3 | CC3/b-actin | CC3/b-actin | Ki67/ b-actin | Fold change 1 | Fold change 2 | Fold change 3 | Average Fold change |
| 0 | 243 | 200 | 182 | 208.3333 | 0 | 450 | 320 | 430 | 1.85 | 1.60 | 2.36 | 1.00 | 1.00 | 1.00 | 1.00 |
| 40 | 216 | 196 | 234 | 215.3333 | 40 | 380 | 180 | 460 | 1.76 | 0.92 | 1.97 | 0.95 | 0.57 | 0.83 | 0.79 |
| 75 | 270 | 260 | 297 | 275.6667 | 75 | 200 | 180 | 270 | 0.74 | 0.69 | 0.91 | 0.40 | 0.43 | 0.39 | 0.41 |

# P27

IVLDCM (μg/ml)

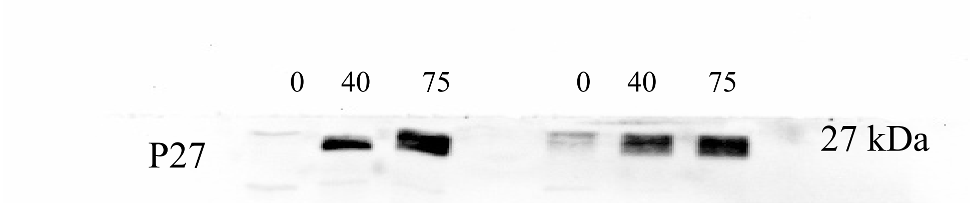


N=1 N=2

| | | | | | | B-actin | | | | | | | | |
|---|---|---|---|---|---|---|---|---|---|---|---|---|---|---|
| P27 | area | area | Fold change | Fold change | Fold change average | Concentration (μg/ml) | Area 1 | Area 2 | P27/b-actin 1 | P27/b-actin 2 | Fold change 1 | Fold change 2 | Fold change average |
| 0 ug/mL | 168 | 231 | 1 | 1 | 1 | 0 | 182 | 200 | 0.92 | 1.16 | 1.00 | 1.00 | 1.00 |
| 40 | 425 | 300 | 2.53 | 1.3 | 1.91 | 40 | 185 | 196 | 2.30 | 1.53 | 2.50 | 1.32 | 1.91 |
| 75 | 456 | 470 | 2.71 | 2.03 | 2.37 | 75 | 210 | 180 | 2.17 | 2.61 | 2.36 | 2.25 | 2.31 |
